# Multidrug resistant yeasts in synanthropic wild birds

**DOI:** 10.1186/1476-0711-9-11

**Published:** 2010-03-23

**Authors:** Alexander Tiong Kah Lord, Kavitha Mohandas, Sushela Somanath, Stephen Ambu

**Affiliations:** 1 The International Medical University, No.126, Jalan 19/155B, Bukit Jalil, 57000 Kuala Lumpur, Malaysia

## Abstract

**Background:**

The aim of this study was to investigate the presence of multidrug resistant yeasts in the faeces of synanthropic wild birds from the Bangsar suburb of Kuala Lumpur.

**Methods:**

Species characterisations of yeast isolates and determinations of antimycotic susceptibility profiles were undertaken using the commercial characterization kit, Integral System Yeasts Plus (Liofilchem, Italy).

**Results:**

Fourteen species of yeasts were detected in the bird faecal samples.*Candida albicans *was present in 28.89% of bird faecal samples, *Candida krusei *(13.33%), *Candida tropicalis *(4.44%), *Candida glabrata *(4.44%), *Candida parapsilosis *(2.22%), *Candida lambica *(2.22%), *Candida stellatoidea *(2.22%), *Candida rugosa *(2.22%) and *Candida lusitaniae *(2.22%). Amongst the non-candidal yeast isolates, *Cryptococcus laurentii *was present in 6.67% of bird faecal samples, *Cryptococcus uniguttulatus *(4.44%), *Saccharomyces cerevisiae *(4.44%), *Trichosporon pullulans *(2.22%), *Trichosporon pullulans/Cryptococcus albidus *(8.89%) and *Rhodotorula rubra/Rhodotorula glutinis *(4.44%). Of the isolated yeasts, 18.1% (or 26/144) were found to be resistant to all 11 antimycotic agents they were tested against i.e. Nystatin, Amphotericin B, Flucytosine, Econazole, Ketoconazole, Clotrimazole, Miconazole, Itraconazole, Voriconazole, Fluconazole 16 and Fluconazole 64. 45.8% (or 66/144) of the bird faecal yeast isolates were resistant to four or more of the 11 antimycotic agents they were tested against.

**Conclusions:**

This finding is of public health significance as these synanthropic wild birds may be reservoirs for transmission of drug resistant yeast infections to humans.

## Background

Wild birds that inhabit urban regions have long been known to harbour human pathogens with zoonotic potential. Amongst reported cases are *Listeria monocytogenes *in wild birds around Helsinki [1], *Chlamydophila psittaci *in pigeons and other free-living species in Zagreb [2], *Campylobacter *spp. in ducks in Washington [3], *Salmonella *spp. in gulls in the Czech Republic [4], pathogenic *Escherichia coli*, *Enterobacter cloacae*, *Salmonella *spp., *Aeromonas hydrophilia *and *Providencia alcalifaciens *in Canada geese found in London parks [5], *Cryptosporidium *spp. and *Giardia *spp. in Canada geese in Maryland [6], *Eimeria magnalabia *(3%), *Eimeria hermani *(14%), *Eimeria truncata *(2%) and *Tyzzeria parvula *in Canada geese in Ontario [7], *Histoplasma capsulatum *in chickens, pigeons, starlings, blackbirds and bats [8] and West Nile Virus in urban birds in Georgia [9]. In Malaysia, a recent study done by Hwee Yong *et al *[10] showed the presence of the Enterobacteriaceae and protozoan parasites in the stool of the large billed Crow (*Corvus *spp.) in Kuala Lumpur. Perhaps the best known example occurring globally is the current situation with Highly Pathogenic Avian Influenza Virus (HPAIV) in poultry and migratory wild birds [11]. As such, although the presence of these birds, upon occasion, is aesthetically pleasing in parks and recreational bodies of water, and without doubt, they do function as pollinators within cities, large numbers when present, particularly in the vicinity of high risk areas such as hospitals, are a potential health hazard.

This study focuses on the Bangsar area, an affluent residential suburb of Kuala Lumpur, the capital city of Malaysia. It is heavily populated and endowed with numerous open air restaurants and road side food stalls. Many of the sampling sites chosen for this study were in close proximity to these eateries and had the potential to become contaminated with pathogens that were harboured in bird faeces that littered the surrounding areas. Bangsar also boasts one major hospital, clinics and other nearby hospitals.

## Methods

### Sample collection

A total of 45 wild bird faecal samples were collected from 12 designated sampling sites from the Bangsar suburb of Kuala Lumpur. Collection of bird faecal samples from these sites was randomised. Fresh faecal samples were collected at two week intervals at 7 pm when the wild birds came to roost for the night. Faecal samples were loaded into eppendorf tubes containing sterile Sabouraud's Dextrose Broth supplemented with 0.5 g/L chloramphenicol (SDB-chmp) to inhibit bacterial growth. Samples were stored in a cooler box and transported to the research laboratory immediately. Upon arrival, samples were weighed, vortexed briefly, then stored at 4°C before processing.

### Viability Assay

Cold stored samples were vortexed and plated onto Sabouraud's Dextrose Agar plates supplemented with 0.5 g/L chloramphenicol (SDA-chmp). Immediately after plating, plates were incubated at 37°C in the dark for five days. On the sixth day of incubation, viable counts were taken. Counts were expressed as ***x ***CFU per milliliter fresh weight faeces.

### Species characterizations and determinations of antimycotic susceptibility profiles

From each of the 45 faecal samples, seven isolates were randomly selected from the SDA-chmp viability assay plates and subcultured twice for purification. Species characterisations of these isolates and determinations of antimycotic susceptibility profiles were undertaken using the commercial characterization kit, Integral System Yeasts Plus (Liofilchem, Italy).

## Results

Fourteen species of yeasts were detected (Additional File 1).*Candida albicans *was present in 28.89% of bird faecal samples, *Candida krusei *(13.33%), *Candida tropicalis *(4.44%), *Candida glabrata *(4.44%), *Candida parapsilosis *(2.22%), *Candida lambica *(2.22%), *Candida stellatoidea *(2.22%), *Candida rugosa *(2.22%) and *Candida lusitaniae *(2.22%). Amongst the non-candidal yeast isolates, *Cryptococcus laurentii *was present in 6.67% of bird faecal samples, *Cryptococcus uniguttulatus *(4.44%), *Saccharomyces cerevisiae *(4.44%), *Trichosporon pullulans *(2.22%), *Trichosporon pullulans/Cryptococcus albidus *(8.89%) and *Rhodotorula rubra/Rhodotorula glutinis *(4.44%).

A significant proportion of the yeasts isolated from the bird faecal samples were found to be drug resistant or multidrug resistant (Additional Files 2, 3, 4, 5, 6, 7, 8, 9, 10, 11, 12, 13, 14, 15, 16). 18.1% (or 26/144) were found to be resistant to all 11 of the antimycotic agents they were tested against. 45.8% (or 66/144) of the bird faecal yeast isolates were resistant to four or more of the 11 antimycotic agents they were tested against (Table 1).

**Table 1:** Proportion and percentage of drug resistant and multidrug resistant bird faecal yeast isolates by genus/species

Genus/Species	Isolates resistant to all 11 antimycotic agents		Isolates resistant to four or more antimycotic agents	
	Proportion	Percentage (%)	Proportion	Percentage (%)
All genera/species	26/144	18.1	66/144	45.8

*Candida*	1/75	1.3	19/75	25.3
*C. albicans*	0/53	0.0	7/53	13.2
*C. parapsilosis*	0/3	0.0	0/3	0.0
*C. tropicalis*	0/2	0.0	2/2	100.0
*C. lambica*	0/1	0.0	0/1	0.0
*C. stellatoidea*	1/1	100.0	1/1	100.0
*C. glabrata*	0/3	0.0	1/3	33.3
*C. krusei*	0/7	0.0	6/7	85.7
*C. rugosa*	0/2	0.0	0/2	0.0
*C. lusitaniae*	0/3	0.0	2/3	66.7

*Cryptococcus*	8/10	80.0	8/10	80.0

*Cryptococcus uniguttulatus*	0/2	0.0	0/2	0.0
*Cryptococcus laurentii*	8/8	100.0	8/8	100.0
*Saccharomyces cerevisiae*	0/5	0	0/5	0.0

*Trichosporon pullulans*	2/2	100	2/2	100.0

*Trichosporon pullulans/Cryptococcus albidus*	7/8	87.5	7/8	87.5

*Rhodotorula rubra/Rhodotorula glutinis*	0/5	0	2/5	40.0

Unidentified	9/39	23.1	27/39	69.2

The following proportions of isolates demonstrated resistance to four or more antimycotic agents: 13.2% (or 7/53) of the *C. albicans *isolates, 33.3% (or 1/3) of the *C. glabrata *isolates, 85.7% (or 6/7) of the *C. krusei *isolates, 66.7% (or 2/3) of the *C. lusitaniae *isolates, all (or 2/2) of the *C. tropicalis *isolates and all (or 1/1) of the *C. stellatoidea *isolates. Of the noncandidal isolates, 100% (or 8/8) of the *Cryptococcus laurentii *isolates, 100% (or 2/2) of the *T. pullulans *isolates, 87.5% (or 7/8) of the *T. pullulans/C. albidus *isolates, 40% (or 2/5) of the *R. rubra/R. glutinis *isolates and 69.2% (or 27/39) of the unidentified yeast isolates were resistant to four or more antimycotic agents.

## Discussion

The findings of this study indicate the presence of multidrug resistant yeasts in the faeces of synanthropic wild birds in Bangsar. Furthermore, many of the species of yeasts detected are documented human pathogens with zoonotic potential [12].

28.89% of the bird faecal samples harboured *C. albicans *and this was the most prevalent species of yeast in the faecal samples. European surveys indicate that this species is responsible for more than half the cases of invasive candidaemia; however, the occurrence of non-albicans related disease appears to be increasing [13]. During the past decade there has been an increasing trend of systemic and fatal infections with non albicans species such as *Candida tropicalis*, *Candida glabrata, Candida parapsilosis*, *Candida lusitaniae *and *Candida lipolytica*. Identification of these species is essential for effective therapy in view of the emergence of resistance to antifungal drugs in these species [14].*Candida parapsilosis *is a major emerging human pathogen that has dramatically increased in significance and prevalence over the past two decades, and is now one of the leading causes of invasive candidal disease. Individuals at the highest risk for severe infection include neonates and patients in intensive care units [15].

Non-neoformans cryptococci have traditionally been thought of as saprophytes but the incidence of human infection with these species has increased with *Cryptococcus laurentii *and *Cryptococcus albidus *together responsible for 80% of non-neoformans reported cases [16]. These species are emerging fungal pathogens to be reckoned with. All eight *C. laurentii *isolates obtained from this study were resistant to all 11 of the antimycotic agents they were tested against. *C. laurentii *has been reported as a rare cause of CAPD associated peritonitis, pulmonary and cutaneous infections [17], fungaemia in a premature neonate [18] and invasive disease in a nine-year-old boy with X-linked hyper-immunoglobulin M syndrome [19]. *C. albidus *has been reported to cause cutaneous infections [20], eye and blood infections in HIV patients [21], pulmonary infection and fungaemia in a leukaemia patient [22] and mucormycosis empyema in a haemodialysis patient [23].

It has been reported that antifungal primary prophylaxis with either itraconazole or fluconazole is effective in reducing the incidence of cryptococcal disease in adults with advanced HIV disease [24]. The results of this study are of concern as the *C. laurentii *isolates obtained are resistant to both itraconazole, fluconazole and all the other antimycotic agents they were tested against.

*Rhodotorula rubra *has been implicated in patients suffering from meningitis and keratitis, albeit in the debilitated and the immunocompromised [25,26]. *Rhodotorula glutinis *on the other hand has been reported to cause meningitis in an immunocompetent patient [27]. *Trichosporon pullulans *has been reported to have caused pulmonary infection in a leukaemia patient [28], infections in a renal transplant patient [29] and in two patients with chronic granulomatous disease [30], amongst others.

Perhaps what may also be significant are the isolates that have not been identified as they were beyond the resolving power of the commercial identification kit used. 69.2% of them were resistant to four or more antimycotic agents. Some of these isolates may be human pathogens or may have the potential of being human pathogens.

Nosocomial fungal infections are seen to be increasingly significant amongst the critically ill. Fungi are now amongst the most frequently isolated organisms in intensive care units. Reports describe potentially fatal fungal infections that are resistant to many commonly used antifungal agents [31], reminiscent of the drug resistant and multidrug resistant yeasts isolated in this study. It is therefore important to recognize and minimize the major risk factors associated with infection, including the existence of synanthropic animal reservoirs. This study supports previous reports that describe the potential of synanthropic birds as carriers, reservoirs and disseminators of pathogens.

In the Bangsar area, the predominant species of bird are wild crows. Not being of the domesticated variety, these birds would not have encountered antifungal chemotherapy. As such, the drug resistant yeasts obtained in this study are either originally human strains that evolved resistance during human therapy with antifungals and subsequently were disseminated to these wild birds, or alternatively, different mechanisms of antifungal resistance development are at work. Possibly, polyspecific transporters, if they exist in these drug resistant yeasts, may have evolved in response to toxins/poisons that are commonly encountered by the birds that harbour these yeasts, either in the wild or in the cities where they frequently dwell. As such, it would be of epidemiological interest to determine whether (1) sylvatic crows living in proximity to Bangsar harbour similarly drug resistant strains of yeasts, and (2) whether the drug resistant yeast strains obtained in this study are the same as that occurring in hospitals and birds associated with hospitals in the vicinity of the study site.

While mass culling and disposal is the well prescribed method of dealing with contagion in domesticated and wild animals, it is expensive, labour-intensive and does not in any way address the root causes of the problem. As such, it is not a long-term solution and when applied, often does not prevent recurrence of the problem. If colonization with these yeasts is directly associated with the immune status of the birds, then the root of the problem lies in the factors that lower bird vitality. The latter include ecological changes that result from urbanization and growth of megacities, and the lack of or breakdown in sanitation that often ensues. Chemical pollution, noise pollution and climate change as a result of shifting temperatures and precipitation levels, are yet other potential contributory factors to colonization and the emergence of disease, by way of modifying both bird and microbial behaviour.

## Conclusions

This study demonstrates that synanthropic wild birds in the Bangsar area harbour multidrug resistant *Cryptococcus laurentii *and other drug resistant and multidrug resistant yeasts. Both the candidal and noncandidal yeast species isolated have been associated with opportunistic and disseminated infections in man, particularly in those with some form of underlying immune suppression, including those on high-dose corticosteroid therapy, having neutropenia or human immunodeficiency virus (HIV) infection [32-35]. In view of the burgeoning immunosuppressed populations both in Malaysia and globally [36,37], the existence and evolution of drug resistant and multidrug resistant strains of yeasts in synanthropic birds that commonly inhabit urban regions, is a source of public health concern.

## Competing interests

The authors declare that they have no competing interests.

## Authors' contributions

Authors' contributions are in the order of listed names. ATKL participated in the study design, carried out the bench work and conducted an extensive literature review. KM and SS participated in the study design and contributed to writing of the manuscript. SA contributed ideas and his broad experience in the field of environmental health. All authors read and approved the final manuscript.

## Supplementary Material

Additional file 1 Chart showing the percentage of wild bird faecal samples that were positive for the genus/species of yeast (n = 45); mean load (m) of each genus/species in positive samples in CFU/ml faeces and range (r) are given in the boxes. Click here for file

Additional file 2 Antimycotic susceptibility profile of *Candida albicans *isolates from bird faeces. Click here for file

Additional file 3 Antimycotic susceptibility profile of *Candida parapsilosis *isolates from bird faeces. Click here for file

Additional file 4 Antimycotic susceptibility profile of *Candida tropicalis *isolates from bird faeces. Click here for file

Additional file 5 Antimycotic susceptibility profile of *Candida lambica *isolates from bird faeces. Click here for file

Additional file 6 Antimycotic susceptibility profile of *Candida stellatoidea *isolates from bird faeces. Click here for file

Additional file 7 Antimycotic susceptibility profile of *Candida glabrata *isolates from bird faeces. Click here for file

Additional file 8 Antimycotic susceptibility profile of *Candida krusei *isolates from bird faeces. Click here for file

Additional file 9 Antimycotic susceptibility profile of *Candida rugosa *isolates from bird faeces. Click here for file

Additional file 10 Antimycotic susceptibility profile of *Candida lusitaniae *isolates from bird faeces. Click here for file

Additional file 11 Antimycotic susceptibility profile of *Cryptococcus uniguttulatus *isolates from bird faeces. Click here for file

Additional file 12 Antimycotic susceptibility profile of *Cryptococcus laurentii *isolates from bird faeces. Click here for file

Additional file 13 Antimycotic susceptibility profile of *Saccharomyces cerevisiae *isolates from bird faeces. Click here for file

Additional file 14 Antimycotic susceptibility profile of *Trichosporon pullulans *isolates from bird faeces. Click here for file

Additional file 15 Antimycotic susceptibility profile of *Rhodotorula *isolates from bird faeces. Click here for file

Additional file 16 Antimycotic susceptibility profile of unidentified yeast isolates from bird faeces. Click here for file
