# Supplementary material for: Multidrug resistant yeasts in synanthropic wild birds
Source: Ann Clin Microbiol Antimicrob. 2010 Mar 23;9:11. doi: 10.1186/1476-0711-9-11 (PMC2852373; doi:10.1186/1476-0711-9-11)
Supplement: Additional file 1 — Chart showing the percentage of wild bird faecal samples that were positive for the genus/species of yeast (n = 45); mean load (m) of each genus/species in positive samples in CFU/ml faeces and range (r) are given in the boxes. [file 1476-0711-9-11-S1.DOC]

**Additional File 1**

**m = 7.36×10^4^**

**r = 2.14×10^2^  - 2.89×10^5^**

**n = 28**

**m = 7.10×10^4^**

**r = 3.72×10^2^**

**- 2.62×10^5^**

**n = 13**

**Load = 1.12×10^3^**

**n = 1**

**m = 2.22×10^3^**

**r = 1.42×10^3^ - 3.02×10^3^**

**n = 2**

**m = 6.67×10^4^**

**r = 2.44×10^3^ - 1.31×10^5^**

**n = 2**

**Load = 3.98×10^2^**

**n = 1**

**Load = 2.14×10^2^**

**n = 1**

**m = 1.31×10^5^**

**r = 5.57×10^2^ - 2.62×10^5^**

**n = 2**

**m = 2.21×10^4^**

**r = 1.99×10^2^- 9.62×10^4^**

**n = 6**

**Load = 7.00×10^3^**

**n = 1**

**Load = 2.89×10^5^**

**n = 1**

**m = 3.43×10^2^**

**r = 3.14×10^2^**

**- 3.72×10^2^**

**n = 2**

**m = 1.49×10^5^**

**r = 1.53×10^3^ - 4.17×10^5^**

**n = 3**

**m = 1.26×10^4^**

**r = 1.53×10^3^ –**

**4.08×10^4^**

**n = 4**

**Load = 1.69×10^5^**

**n = 1**

**m = 2.29×10^4^**

**r = 3.50×10^3^ - 4.23×10^4^**

**n = 2**
