# Supplementary material for: Multidrug resistant yeasts in synanthropic wild birds
Source: Ann Clin Microbiol Antimicrob. 2010 Mar 23;9:11. doi: 10.1186/1476-0711-9-11 (PMC2852373; doi:10.1186/1476-0711-9-11)
Supplement: Additional file 2 — Antimycotic susceptibility profile of Candida albicans isolates from bird faeces. [file 1476-0711-9-11-S2.DOC]

**Key to Tables 1 –15**

**R = resistant** ; **I = intermediate** ; **S = susceptible**

**NY: Nystatin AMB : Amphotericin B FCY : Flucytosine**

**ECN : Econazole KCA : Ketoconazole CLO : Clotrimazole**

**MIC : Miconazole ITR : Itraconazole VOR : Voriconazole**

**FLU-16 : Fluconazole 16 FLU-64 : Fluconazole 64**

**Table 1 Antimycotic susceptibility profile of *Candida albicans* isolates from bird faeces**

| **Species** | **Code** | **NY** | **AMB** | **FCY** | **ECN** | **KCA** | **CLO** | **MIC** | **ITR** | **VOR** | **FLU-16** | **FLU-64** |
| --- | --- | --- | --- | --- | --- | --- | --- | --- | --- | --- | --- | --- |
| ***C. albicans*** | **S3b** | **S** | **S** | **S** | **S** | **S** | **S** | **S** | **S** | **S** | **S** | **S** |
|  | **S5a** | **S** | **S** | **S** | **S** | **S** | **S** | **S** | **S** | **S** | **S** | **S** |
|  | **S5b** | **S** | **S** | **S** | **S** | **S** | **S** | **S** | **S** | **S** | **S** | **S** |
|  | **S5c** | **S** | **S** | **S** | **S** | **S** | **S** | **S** | **S** | **S** | **S** | **S** |
|  | **S5d** | **S** | **S** | **S** | **S** | **S** | **S** | **S** | **S** | **S** | **S** | **S** |
|  | **S5e** | **S** | **S** | **S** | **S** | **S** | **S** | **S** | **S** | **S** | **S** | **S** |
|  | **S7c** | **S** | **S** | **S** | **S** | **S** | **I** | **S** | **I** | **I** | **I** | **I** |
|  | **S7d** | **S** | **S** | **S** | **I** | **S** | **I** | **I** | **I** | **I** | **I** | **I** |
|  | **S8a** | **S** | **S** | **S** | **I** | **S** | **I** | **S** | **S** | **S** | **S** | **S** |
|  | **S8c** | **S** | **S** | **S** | **S** | **S** | **S** | **I** | **S** | **S** | **S** | **S** |
|  | **S8d** | **S** | **S** | **S** | **I** | **S** | **I** | **I** | **S** | **S** | **S** | **S** |
|  | **S8e** | **S** | **S** | **S** | **I** | **S** | **I** | **I** | **S** | **S** | **S** | **S** |
|  | **S14b** | **R** | **S** | **S** | **I** | **S** | S | S | S | **I** | S | S |
|  | **S14c** | **R** | **S** | **S** | **S** | **S** | **S** | **S** | **S** | **S** | **S** | **S** |
|  | **S14d** | **I** | **S** | **S** | **S** | **S** | **S** | **S** | **S** | **S** | **S** | **S** |
|  | **S14e** | **R** | **S** | **S** | **S** | **S** | **I** | **I** | **I** | **I** | **I** | **S** |
|  | **S14f** | **R** | **S** | **S** | **I** | **I** | **S** | **S** | **S** | **I** | **S** | **S** |
|  | **S14g** | **R** | **S** | **S** | **R** | **R** | **S** | **S** | **S** | **S** | **S** | **S** |
|  | **S25a** | **S** | **S** | **R** | **S** | **S** | **I** | **S** | **S** | **S** | **R** | **S** |
|  | **S25b** | **S** | **S** | **R** | **S** | **S** | **S** | **S** | **S** | **S** | **S** | **S** |
|  | **S25c** | **I** | **S** | **R** | **S** | **S** | **I** | **S** | **S** | **S** | **S** | **S** |
|  | **S25d** | **I** | **S** | **I** | **S** | **S** | **S** | **S** | **S** | **S** | **S** | **S** |
|  | **S25e** | **R** | **S** | **R** | **S** | **S** | **S** | **R** | **S** | **S** | **I** | **S** |
|  | **S26a** | **S** | **S** | **S** | **S** | **S** | **S** | **S** | **S** | **S** | **S** | **S** |
|  | **S26b** | **S** | **S** | **S** | **S** | **S** | **S** | **S** | **S** | **S** | **S** | **S** |
|  | **S26c** | **S** | **S** | **S** | **S** | **S** | **S** | **S** | **S** | **S** | **S** | **S** |
|  | **S26d** | **I** | **S** | **S** | **S** | **S** | **S** | **S** | **S** | **S** | **S** | **S** |
|  | **S29a** | **I** | **S** | **S** | **R** | **S** | **S** | **S** | **I** | **I** | **I** | **I** |
|  | **S29c** | **S** | **S** | **S** | **R** | **S** | **R** | **R** | **I** | **R** | **R** | **R** |
|  | **S29d** | **I** | **S** | **S** | **S** | **S** | **S** | **S** | **S** | **S** | **S** | **S** |
|  | **S29e** | **S** | **S** | **I** | **I** | **S** | **R** | **R** | **I** | **R** | **I** | **R** |
|  | **S36a** | **I** | **S** | **S** | **I** | **S** | **S** | **S** | **S** | **S** | **S** | **S** |
|  | **S36b** | **I** | **S** | **S** | **S** | **S** | **S** | **S** | **S** | **S** | **S** | **S** |
|  | **S36c** | **I** | **S** | **S** | **S** | **S** | **S** | **S** | **S** | **S** | **S** | **S** |
|  | **s36d** | **I** | **S** | **S** | **S** | **S** | **S** | **S** | **S** | **S** | **S** | **S** |
|  | **S36e** | **I** | **S** | **S** | **S** | **S** | **S** | **S** | **S** | **S** | **S** | **S** |
|  | **S39a** | **I** | **S** | **S** | **S** | **S** | **S** | **S** | **S** | **S** | **S** | **S** |
|  | **S39b** | **I** | **S** | **S** | **S** | **S** | **S** | **S** | **S** | **S** | **S** | **S** |
|  | **S39c** | **I** | **S** | **S** | **S** | **S** | **S** | **S** | **S** | **S** | **S** | **S** |
|  | **S39d** | **S** | **S** | **S** | **S** | **S** | **S** | **S** | **S** | **S** | **S** | **S** |
|  | **S39e** | **S** | **S** | **S** | **S** | **S** | **S** | **S** | **S** | **S** | **S** | **S** |
|  | **S40a** | **R** | **I** | **S** | **R** | **S** | **R** | **R** | **R** | **R** | **R** | **R** |
|  | **S40b** | **I** | **S** | **S** | **R** | **R** | **R** | **R** | **R** | **R** | **R** | **R** |
|  | **S40c** | **S** | **S** | **S** | **R** | **S** | **R** | **R** | **R** | **I** | **I** | **R** |
|  | **S40d** | **S** | **S** | **S** | **R** | **S** | **I** | **I** | **I** | **R** | **S** | **I** |
|  | **S40e** | **S** | **S** | **S** | **R** | **S** | **R** | **R** | **R** | **R** | **R** | **R** |
|  | **S42b** | **S** | **S** | **S** | **I** | **S** | **I** | **R** | **S** | **S** | **S** | **S** |
|  | **S42c** | **I** | **S** | **S** | **R** | **S** | **R** | **R** | **R** | **R** | **R** | **R** |
|  | **S44a** | **I** | **S** | **S** | **S** | **S** | **S** | **S** | **S** | **S** | **S** | **S** |
|  | **S44b** | **S** | **S** | **S** | **S** | **S** | **S** | **S** | **S** | **S** | **S** | **S** |
|  | **S44c** | **S** | **S** | **S** | **S** | **S** | **S** | **S** | **S** | **S** | **S** | **S** |
|  | **S44d** | **S** | **S** | **S** | **S** | **S** | **S** | **S** | **S** | **S** | **S** | **S** |
|  | **S44e** | **I** | **S** | **S** | **S** | **S** | **S** | **S** | **S** | **S** | **S** | **S** |
