# Supplementary material for: Multidrug resistant yeasts in synanthropic wild birds
Source: Ann Clin Microbiol Antimicrob. 2010 Mar 23;9:11. doi: 10.1186/1476-0711-9-11 (PMC2852373; doi:10.1186/1476-0711-9-11)
Supplement: Additional file 6 — Antimycotic susceptibility profile of Candida stellatoidea isolates from bird faeces. [file 1476-0711-9-11-S6.DOC]

**Key to Tables 1 –15**

**R = resistant** ; **I = intermediate** ; **S = susceptible**

**NY: Nystatin AMB : Amphotericin B FCY : Flucytosine**

**ECN : Econazole KCA : Ketoconazole CLO : Clotrimazole**

**MIC : Miconazole ITR : Itraconazole VOR : Voriconazole**

**FLU-16 : Fluconazole 16 FLU-64 : Fluconazole 64**

**Table 5 Antimycotic susceptibility profile of *Candida stellatoidea* isolates**

**from bird faeces**

| **Species** | **Code** | **NY** | **AMB** | **FCY** | **ECN** | **KCA** | **CLO** | **MIC** | **ITR** | **VOR** | **FLU-16** | **FLU-64** |
| --- | --- | --- | --- | --- | --- | --- | --- | --- | --- | --- | --- | --- |
| ***C. stellatoidea*** | **S12g** | **R** | **R** | **g** | **R** | **R** | **R** | **R** | **R** | **R** | **R** | **R** |
