# Supplementary material for: Multidrug resistant yeasts in synanthropic wild birds
Source: Ann Clin Microbiol Antimicrob. 2010 Mar 23;9:11. doi: 10.1186/1476-0711-9-11 (PMC2852373; doi:10.1186/1476-0711-9-11)
Supplement: Additional file 16 — Antimycotic susceptibility profile of unidentified yeast isolates from bird faeces. [file 1476-0711-9-11-S16.DOC]

**Key to Tables 1 –15**

**R = resistant** ; **I = intermediate** ; **S = susceptible**

**NY: Nystatin AMB : Amphotericin B FCY : Flucytosine**

**ECN : Econazole KCA : Ketoconazole CLO : Clotrimazole**

**MIC : Miconazole ITR : Itraconazole VOR : Voriconazole**

**FLU-16 : Fluconazole 16 FLU-64 : Fluconazole 64**

**Table 15 Antimycotic susceptibility profile of unidentified yeast isolates from bird faeces**

| **Species** | **Code** | **NY** | **AMB** | **FCY** | **ECN** | **KCA** | **CLO** | **MIC** | **ITR** | **VOR** | **FLU-16** | **FLU-64** |
| --- | --- | --- | --- | --- | --- | --- | --- | --- | --- | --- | --- | --- |
| ***Unknown*** | **S8g** | **S** | **S** | **R** | **S** | **S** | **R** | **R** | **S** | **I** | **R** | **R** |
|  | **S12b** | **R** | **R** | **S** | **R** | **R** | **R** | **R** | **R** | **R** | **R** | **R** |
|  | **S12c** | **R** | **R** | **S** | **R** | **R** | **R** | **R** | **R** | **R** | **R** | **R** |
|  | **S12d** | **R** | **R** | **S** | **R** | **R** | **R** | **R** | **R** | **R** | **R** | **R** |
|  | **S12f** | **R** | **R** | **S** | **R** | **R** | **R** | **R** | **R** | **R** | **R** | **R** |
|  | **S2a** | **S** | **S** | **S** | **I** | **S** | **R** | **R** | **S** | **R** | **I** | **S** |
|  | **S2b** | **S** | **S** | **S** | **I** | **S** | **R** | **R** | **S** | **R** | **I** | **S** |
|  | **S2c** | **S** | **S** | **S** | **R** | **S** | **R** | **R** | **S** | **R** | **I** | **S** |
|  | **S2d** | **S** | **S** | **S** | **I** | **S** | **I** | **R** | **I** | **R** | **R** | **R** |
|  | **S4b** | **I** | **I** | **S** | **I** | **S** | **I** | **R** | **I** | **I** | **S** | **S** |
|  | **S15a** | **R** | **R** | **S** | **R** | **R** | **R** | **R** | **R** | **R** | **R** | **R** |
|  | **S20b** | **S** | **S** | **S** | **I** | **S** | **R** | **I** | **S** | **S** | **S** | **S** |
|  | **S20f** | **S** | **S** | **S** | **S** | **S** | **I** | **S** | **S** | **S** | **S** | **S** |
|  | **S24c** | **R** | **R** | **R** | **R** | **R** | **R** | **R** | **R** | **R** | **R** | **R** |
|  | **S24e** | **S** | **S** | **S** | **R** | **R** | **S** | **R** | **S** | **R** | **R** | **R** |
|  | **S26e** | **S** | **R** | **S** | **R** | **R** | **R** | **S** | **S** | **R** | **R** | **R** |
|  | **S27b** | **R** | **R** | **R** | **R** | **R** | **R** | **R** | **R** | **R** | **R** | **R** |
|  | **S31a** | **S** | **S** | **S** | **R** | **S** | **S** | **S** | **R** | **S** | **S** | **S** |
|  | **S31b** | **S** | **S** | **S** | **S** | **R** | **S** | **S** | **S** | **R** | **S** | **S** |
|  | **S31c** | **S** | **S** | **S** | **S** | **S** | **R** | **S** | **S** | **S** | **S** | **S** |
|  | **S31d** | **S** | **R** | **S** | **S** | **R** | **S** | **S** | **R** | **S** | **S** | **S** |
|  | **S31e** | **S** | **S** | **S** | **I** | **S** | **S** | **S** | **S** | **S** | **S** | **S** |
|  | **S32b** | **S** | **S** | **S** | **S** | **S** | **R** | **S** | **S** | **S** | **S** | **S** |
|  | **S32d** | **S** | **S** | **S** | **R** | **S** | **R** | **R** | **S** | **R** | **R** | **S** |
|  | **S34d** | **R** | **I** | **S** | **R** | **S** | **R** | **R** | **S** | **R** | **S** | **I** |
|  | **S35c** | **R** | **S** | **S** | **R** | **S** | **R** | **R** | **S** | **R** | **R** | **R** |
|  | **S37b** | **R** | **R** | **R** | **R** | **R** | **R** | **R** | **R** | **R** | **R** | **R** |
|  | **S37c** | **R** | **R** | **R** | **R** | **R** | **R** | **R** | **R** | **R** | **R** | **R** |
|  | **S37d** | **R** | **R** | **R** | **R** | **R** | **R** | **R** | **R** | **R** | **R** | **R** |
|  | **S38a** | **R** | **R** | **R** | **R** | **R** | **R** | **R** | **R** | **R** | **R** | **R** |
|  | **S38b** | **R** | **R** | **R** | **R** | **R** | **R** | **R** | **R** | **R** | **R** | **R** |
|  | **S38c** | **R** | **R** | **R** | **R** | **R** | **R** | **R** | **R** | **R** | **R** | **R** |
|  | **S38d** | **R** | **R** | **R** | **R** | **R** | **R** | **R** | **R** | **R** | **R** | **R** |
|  | **S38e** | **R** | **R** | **R** | **R** | **R** | **R** | **R** | **R** | **R** | **R** | **R** |
|  | **S43a** | **S** | **S** | **R** | **S** | **R** | **S** | **R** | **S** | **S** | **R** | **R** |
|  | **S43b** | **S** | **R** | **S** | **S** | **R** | **R** | **S** | **S** | **R** | **R** | **R** |
|  | **S43c** | **S** | **R** | **S** | **R** | **R** | **R** | **R** | **S** | **R** | **R** | **R** |
|  | **S43d** | **S** | **S** | **S** | **S** | **R** | **R** | **S** | **S** | **R** | **R** | **R** |
|  | **S43e** | **R** | **R** | **R** | **R** | **R** | **R** | **R** | **R** | **R** | **R** | **R** |
